# Crosstalk between androgen and Wnt/β-catenin leads to changes of wool density in FGF5-knockout sheep

**DOI:** 10.1038/s41419-020-2622-x

**Published:** 2020-05-29

**Authors:** Rui Zhang, Yan Li, Kun Jia, Xueling Xu, Yao Li, Yue Zhao, Xiaosheng Zhang, Jinlong Zhang, Guoshi Liu, Shoulong Deng, Zhengxing Lian

**Affiliations:** 1 Beijing Key Laboratory of Animal Genetic Improvement, National Engineering Laboratory for Animal Breeding, Key Laboratory of Animal Genetics and Breeding of the Ministry of Agriculture, College of Animal Science and Technology, China Agricultural University, Beijing, 100193 China; 2 Tianjin Institute of Animal Sciences, Tianjin, 300112 China; 3 CAS Key Laboratory of Genome Sciences and Information, Beijing Institute of Genomics, Chinese Academy of Sciences, Beijing, 100101 China

**Keywords:** Genetic engineering, Extracellular signalling molecules

## Abstract

*Fibroblast growth factor 5* (*FGF5*) is a famous dominant inhibitor of anagen phase of hair cycle. Mutations of *FGF5* gene result in a longer wool in mice, donkeys, dogs, cats, and even in human eyelashes. Sheep is an important source of wool production. How to improve the production of wool quickly and effectively is an urgent problem to be solved. In this study, we generated five *FGF5*-knockout Dorper sheep by the CRISPR/Cas9 system. The expression level of *FGF5* mRNA in knockout (KO) sheep decreased significantly, and all FGF5 proteins were dysfunctional. The KO sheep displayed a significant increase in fine-wool and active hair-follicle density. The crosstalk between androgen and *Wnt/β-catenin* signaling downstream of *FGF5* gene plays a key role. We established downstream signaling cascades for the first time, including *FGF5*, *FGFR1*, androgen, *AR*, *Wnt/β-catenin*, *Shh/Gli2*, *c-MYC*, and *KRTs*. These findings further improved the function of *FGF5* gene, and provided therapeutic ideas for androgen alopecia.

## Introduction

Hair is a primary characteristic of mammals. Hair-follicle development takes place during fetal skin development, and relies on tightly regulated ectodermal–mesodermal interactions. After birth, mature and actively growing hair follicles eventually become anchored in the subcutis, and periodically regenerate by spontaneously undergoing repetitive cycles. The hair-growth cycle in mammals is composed of three phases: anagen, catagen, and telogen^1–4^. Many of the signaling cascades involved in embryonic hair development are redeployed during adult hair development, and hyperactivation or inappropriate maintenance of these signaling pathways can result in skin disorders and cancers.

The *FGF5* gene is a member of the *FGF* family, which exhibits a diverse array of biological activities^5^. Hebert et al.^6^ reported that deletion of the *FGF5* gene abnormally prolongs anagen in mice, suggesting that its expression leads to termination of anagen and induction of catagen. Subsequent to identification of *FGF5* as causative for the angora mouse phenotype, genetic variants in *FGF5* have been shown to underlie hair-length regulation in several other species, including cats (*Felis catus*)^7,8^, dogs (*Canis lupus familiaris*)^9,10^, donkeys (*Equus africanus asinus*)^11^, Syrian hamster (*Mesocricetus auratus*)^12^, domestic guinea pigs (*Cavia porcellus*)^13^, and even human (*Homo sapiens*)^14^.

Sheep are one of the most important wool animals. However, traditional breeding limits the genetic improvement of sheep breeding in the short term. Current results indicate that the CRISPR–Cas9 system can be successfully employed in a broad range of organisms^15–21^. In the previous study, we had successfully generated *FGF5*-knockout heterozygous sheep using the CRISPR/Cas9 system by direct one-step cytoplasmic injection of Cas9 mRNA and sgRNA into zygotes, and the relationship between *FGF5* gene and wool length in sheep was proved^22^. In this study, we continue the previous approach, and found that *FGF5* is also related to the wool and active hair-follicle density in Dorper sheep. However, the specific mechanism of the FGF5 gene in the development of hair follicles is unclear, and whether it has other effects besides promoting changes in the follicular cycle is currently elusive.

Androgenetic alopecia (AGA) is the most common form of hair loss in humans, which is mediated mainly by androgens. Androgens regulate hair growth, sebum production, and secretion, among other physiological effects in the skin^23,24^. Androgen levels are under the control of enzymes. Testosterone, as one of the androgens, can be reduced to dihydrotestosterone (DHT) by 5α-reductase (SRD5A) enzyme, which has three isotypes. SRD5A1 is predominantly expressed in skin and annexes^23,25,26^. Hydroxysteroid 17-beta-dehydrogenase 2 (HSD17β2) is also a key player in the inactivation of testosterone^27^. The importance of the *Wnt/β-catenin* pathway in AGA is emphasized by the demonstration of molecular crosstalk between androgens and *Wnt* signaling in dermal papilla cell (DPC)^28^. Androgen/AR complex binding to antioxidative response elements (AREs) containing promoters of target genes disrupts Wnt agonist/antagonist balance involved in DPC-inductive ability, such as dickkopf-1 (DKK1), which is a specific inhibitor of Wnt coreceptors of the LRP family^29^.

*Sonic hedgehog* (*Shh*) also plays an important role in both embryonic and adult hair development. Lim et al.^30^ showed that hair-follicle activation in *Wnt*-active dermal cells promotes their fate conversion into dermal papilla (DP), the regenerative dermal niche for hair-follicle formation. Also, Veltri et al.^31^ believed that *Shh* signaling serves as a downstream pathway of *Wnt/β-catenin* signaling to regulate hair-follicle (HF) induction.

Is there an association between the increase of wool and active hair-follicle density in *FGF5* KO sheep and AGA? If so, does crosstalk between AR and Wnt/β-catenin also participate in the process of wool and active hair-follicle density in *FGF5* KO sheep? Are there any other signaling pathways or other factors that also play a role? In this study, we revealed that the crosstalk between androgen and *Wnt/β-catenin* signaling plays a major role in the increase in wool and active hair-follicle density due to the activation of *c-MYC* and *KRTs* associated with inner root sheath (IRS) in *FGF5* KO sheep, and the *Shh* pathway is also involved in this process as a downstream pathway of *Wnt/β-catenin* signaling.

## Results

### Generation and screening of FGF5 KO sheep

**Fig. 1:**
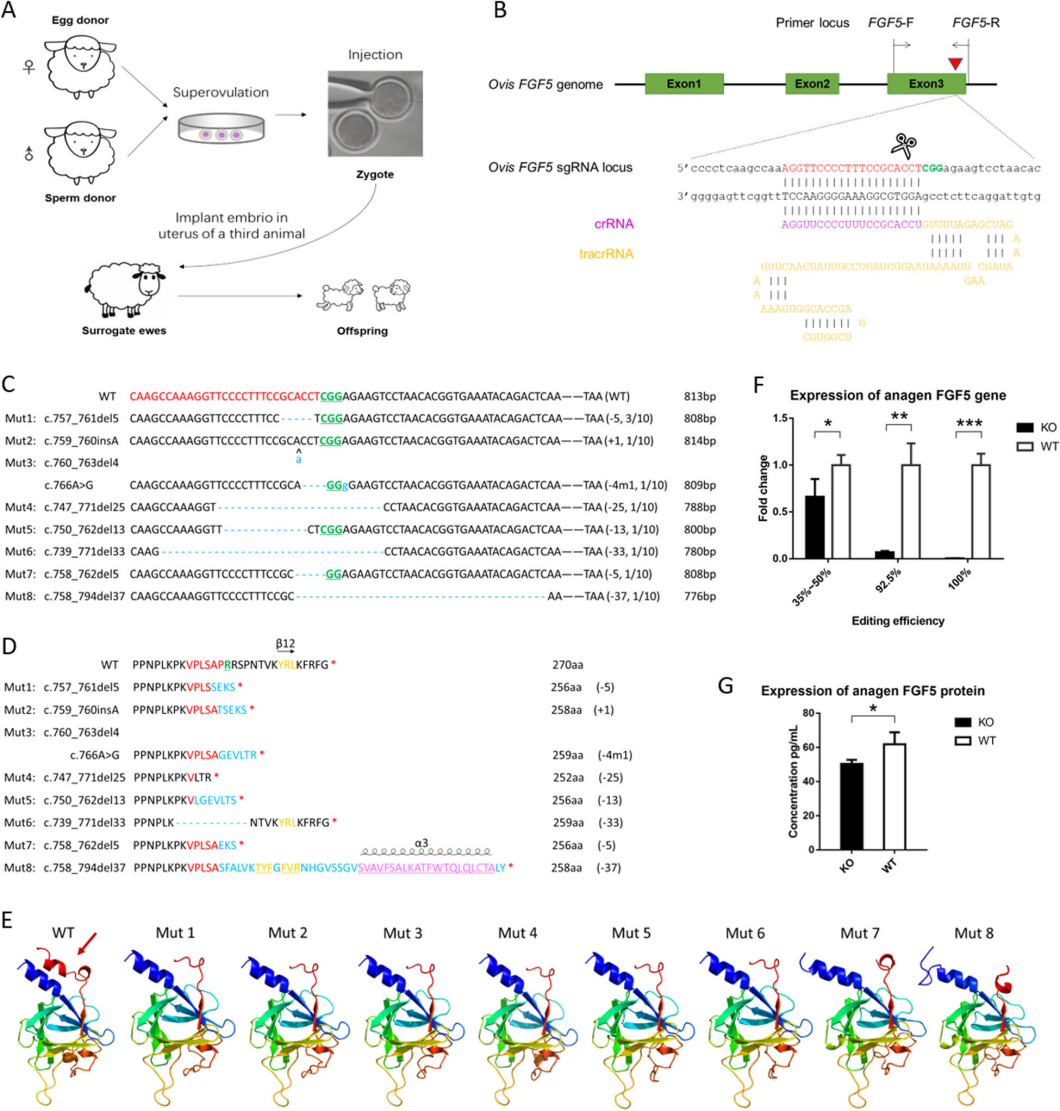
Generation and screening of FGF5-knockout sheep. **a** Representative schematic of the experimental design. After mating and superovulation of the donor sheep, Cas9 mRNA and sgRNA were co-injected into one-cell embryos; then the embryo was implanted into the uterus of a third animal. The editing efficiencies were detected after the birth of the lambs. **b** The targeted sequence and the detecting primer sequences at sheep FGF5 locus. Red triangle indicates the predicted DSB cleavage site for the sgRNA. The PAM and protospacer sequences are highlighted in green and red, respectively. **c** Schematic diagram of the modified FGF5 partial protein-coding region and the targeting locus of sgRNA: Cas9. sgRNA-targeting sites are presented in red text; PAM sequences are highlighted in green and underlined; the mutations are blue, lower case; insertions (+), deletions (−), mutation (m), and the frequencies occurring in individuals are shown to the right of each allele. **d** Schematic diagram of the changes in partial protein AA sequences of modified FGF5 in KO sheep. Protein AA sequences of the sgRNA-targeting site are presented in red text; protein AA sequences of PAM sequence are highlighted in green and underlined; the deletions and changes in protein AA sequences are highlighted in blue; the secondary structure of β-strands is highlighted in yellow, and changes in β-strands caused by mutations are underlined; insertions (+), deletions (−), and mutation (m) are shown to the right of each allele. **e** Changes in the tertiary structure of different mutants. The mutated FGF5 lacked the β12 strands (marked in dark-red arrows). **f** The RNA expression of FGF5 decreases in KO individuals with different editing efficiencies in anagen. **g** The protein expression of FGF5 in the KO group also decreases compared with the control group in anagen by ELISA.

Since we have investigated the efficiency of detecting mutations by PCR sequencing (Supplementary Table S1), the blood DNA template was screened for positive individuals using PCR and DNA sequencing (Supplementary Fig. S1). As expected, there were a total of eight mutants in five founders (including three females and two males) (Fig. 1c), and one mutation appeared in three of the founders.

Cas9 may induce a troublesome off-target effect^32^. A total of 12 most potential off-target sites were predicted across the sheep genome. The sequencing results indicated that there was no detectable indel at on-target sites (Supplementary Fig. S2).

### Structure of FGF5 protein and expression level have changed resulting from gene editing

Sequencing results demonstrated that mosaicism existed in four founders. The indels result in a FGF5 protein frameshift or deletion of AA (Fig. 1d), and the β-12 strands were destroyed or changed. The 3D models also predicted that the mutated FGF5 lacked the β-12 strands that define the canonical trefoil of the FGF family (Fig. 1e).

The results of the transcription and the protein level of *FGF5* expression demonstrated that intact *FGF5* mRNA was significantly downregulated (*P* < 0.01) in the KO group compared with the control group (Figs. 1f and 3b); the intact FGF5 protein was significantly downregulated (*P* < 0.05) as well (Fig. 1g).

### Wool and active follicle density have increased due to gene editing

**Fig. 2:**
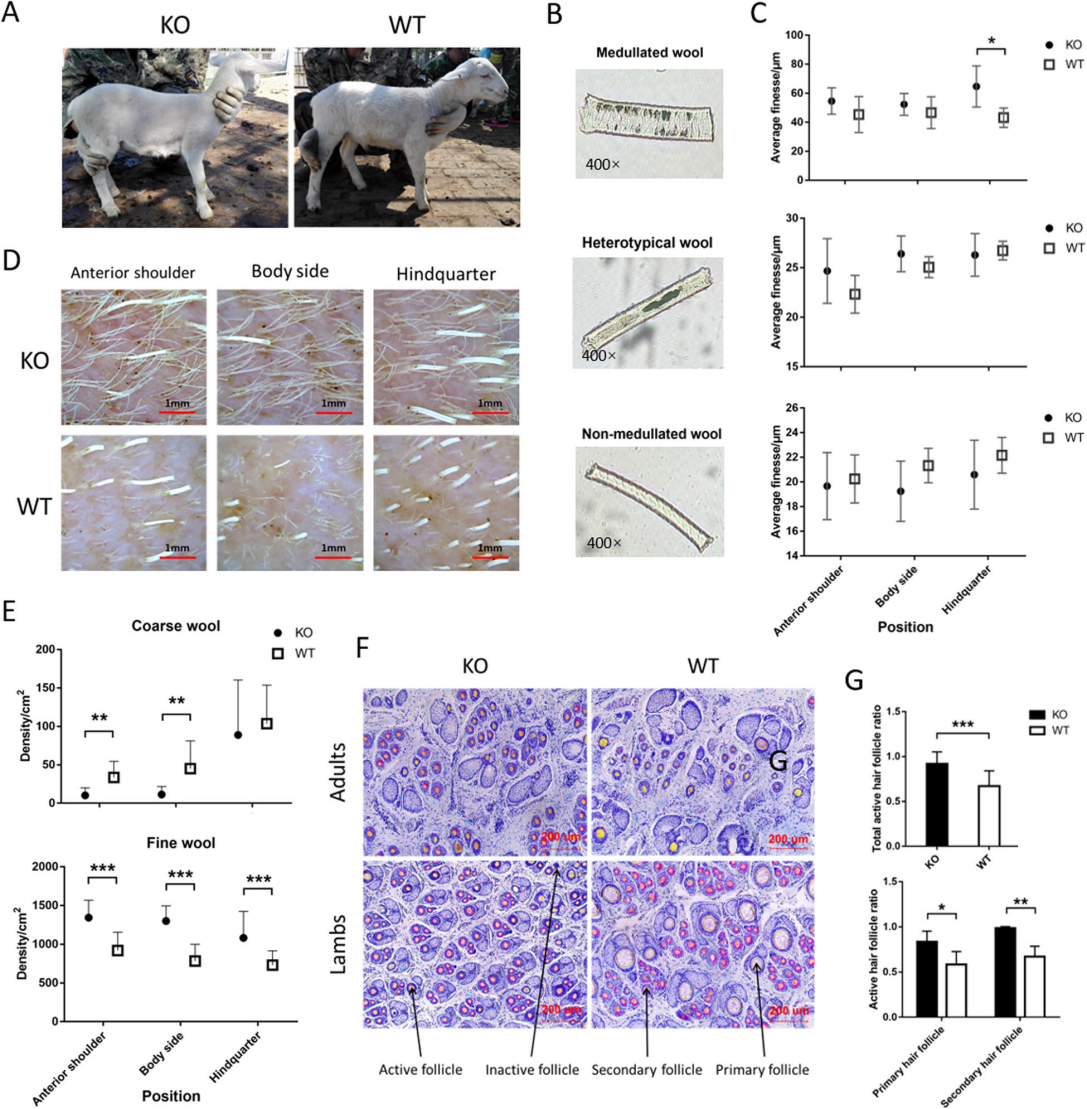
Changes in sheep wool and hair-follicle density after FGF5 gene editing in anagen. **a** Photographs of one of the KO sheep and its sibling. **b** There are three types of wool in the KO and the control group. **c** The changes in fineness of three types of wool in three different parts of KO and control group. Except for the medullated wool of the KO group at the hindquarter, the fineness of which was significantly higher than the control group, there was no significant difference in the fineness of the wools in the other parts. **d** Photograph of skin follicles at 48 h after shaving. The actual area shown in the picture is 16 mm². **e** Changes in coarse- and fine-wool density in the three parts of the KO and control group. The densities of fine wool in the KO group were significantly higher than that in the control group at all three sites. **f** Sacpic staining of adult sheep and lamb skin sections in the KO and control group. The ruler is marked in red in the lower-right corner of the picture. Primary, secondary, active, and inactive hair follicles are also labeled with arrows, respectively. **g** Changes in the total active hair-follicle ratio of coarse and fine wool between the KO and control group. Regardless of the total active hair-follicle ratio or the proportion of active primary and secondary hair follicles, the KO group was significantly higher than the control group. The statistical results in panel **e** are from **d**, and the statistical results in panel **g** are from **f**.

To investigate the changes in wool phenotype caused by the loss-of-function mutation in *FGF5*, the density and fineness of the wool were measured from three parts of both the KO and control group (Fig. 2a). Both the KO and the control group have three types of wool hair, including medullated wool, heterotypical wool, and nonmedullated wool, and the fineness of three types of wool hair has no significant change, except for the medullated wool in the hindquarter (*P* < 0.01, Fig. 2b, c). The coarse-wool densities of the KO group were significantly lower than the control group in the anterior shoulder and body side, while the fine-wool densities of the KO group were significantly higher than the control group in all three parts (*P* < 0.001, Fig. 2d, e). Sacpic staining showed that the proportions of total, primary, and secondary active hair follicles in the KO group, were significantly higher than that in the control group. (Fig. 2f, g). Changes in sheep wool and active hair-follicle density after *FGF5* gene editing in catagen and telogen were also investigated (Supplementary Fig. S3). In catagen, the coarse-wool densities have no significant change, while the fine-wool densities of the KO group were significantly higher than the control group in all three parts (*P* < 0.05, Fig. S3a–c). Sacpic staining results also showed that the proportion of primary active hair follicles has no significant change, while the proportion of secondary active hair follicles in the KO group was significantly higher than that in the control group. In telogen, the densities of coarse and fine wool have no significant change, except that the fine-wool density in the KO group was significantly higher in the anterior shoulder than that in the control group (*P* < 0.01). Sacpic staining results also showed that the proportion of primary and secondary active hair follicles has no significant change.

### Androgen receptor and Wnt signaling operate as key stimulators of HF development in FGF5 KO sheep

**Fig. 3:**
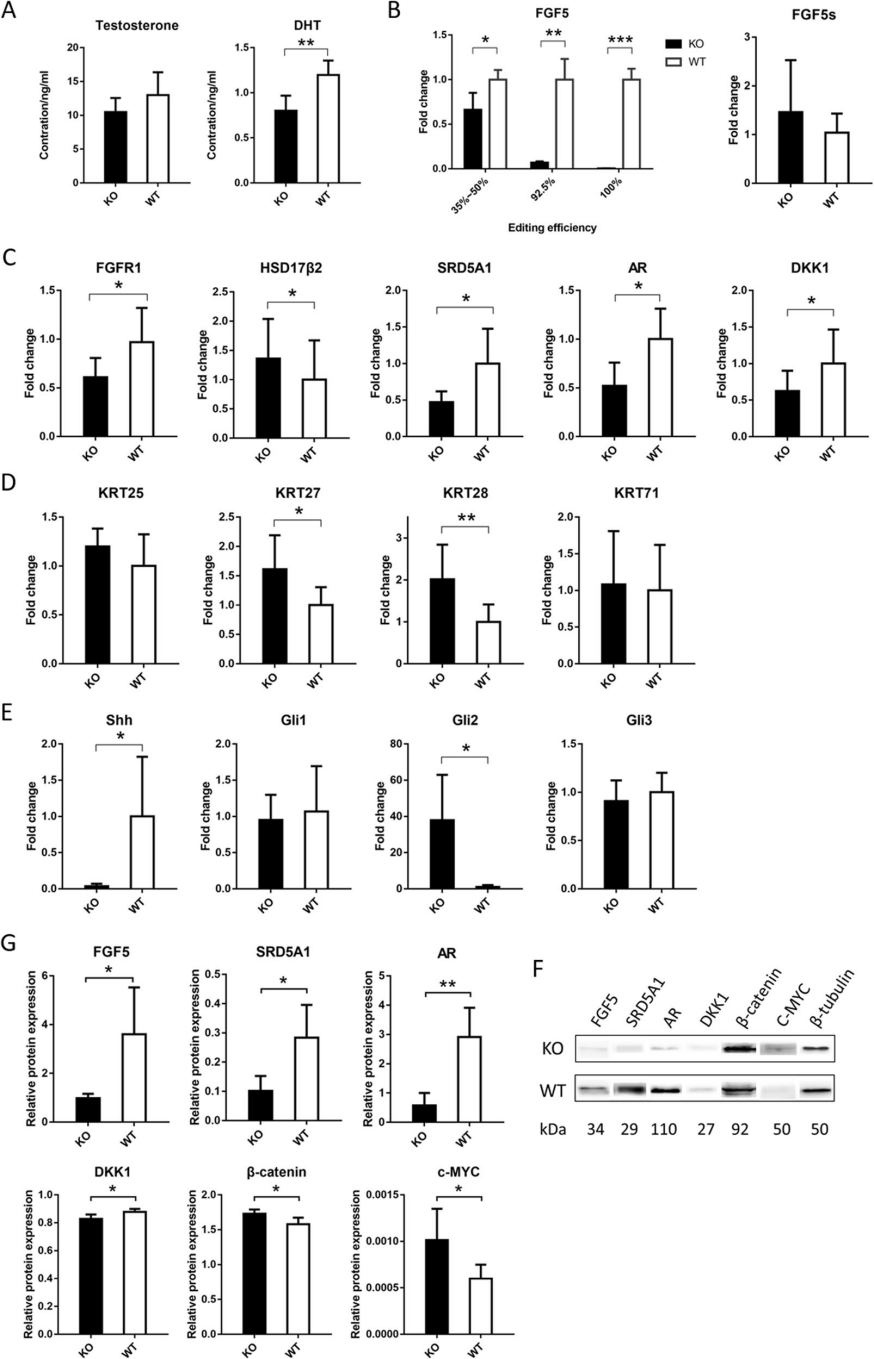
*FGF5*-mediated multiple signaling promotes the increase in wool density and the active hair-follicle density in anagen. **a** Changes in testosterone and DHT in the skin tissue of KO and control groups. The expression level of DHT in the skin tissue of the KO group was significantly lower than that of the control group. **b** Changes in FGF5 in the skin tissue of the KO and control group. The expression levels of FGF5 gene in different editing efficiency individuals were different, but they were significantly lower than the control group. However, the expression level of the FGF5s gene did not change significantly. **c** Expression changes of the related genes in *AR* and *Wnt* signaling in the skin tissue of the KO and control group. **d** Expression changes of IRS-related *KRTs* in the skin tissue of the KO and control group. **e** Expression changes of *Shh* signaling in the skin tissue of the KO and control group. **f** The expression levels of FGF5, SRD5A1, AR, DKK1, β-catenin, and c-MYC proteins in the skin tissue were detected by western blotting. The amount of β-tubulin was used as a control. **g** Compared with the control group, the protein expression levels of FGF5, SRD5A1, AR, DKK1, β-catenin, and c-MYC in the KO group were significantly changed.

To explore the causes of increased wool and active hair-follicle density in *FGF5* KO sheep, we tested the expression levels of testosterone and DHT that cause androgenetic alopecia in the skin tissue of the KO and control groups by ELISA. As a result, the levels of testosterone in the KO and control group were comparable, while the level of DHT was significantly reduced in the KO group (Fig. 3a). The mRNA and the protein levels of SRD5A1, AR, and DKK1 were significantly lower in the KO than in the control group (*P* < 0.05), while *17β-HSD2* mRNA level was significantly higher in the KO group (Fig. 3c). Besides, the protein level of β-catenin was indeed significantly higher in the KO group (Fig. 3f, g). At this point, we were sure that the crosstalk between androgen and *Wnt/β-catenin* signaling was involved in this process.

Based on these results, we have also explored the pathways downstream of *Wnt/β-catenin* signaling. As a result, the expression levels of *KRT27* and *KRT28*, which are essential for the proper assembly of type I and II keratin protein complexes and formation of keratin intermediate filaments in the IRS, were significantly higher in the KO group (Fig. 3d). The expression level of c-MYC was significantly higher in the KO group (Fig. 3f, g). The expression levels of other related genes are shown in Supplementary Fig. S4. In the Wnt signaling pathway, expression levels of *Wnt-10a*, *Wnt-10b*, *LRP6*, *Lef1*, and *β-catenin* were comparable in the KO and control group. The same as related factors in the Wnt signaling pathway, expression levels of related factors in the BMP signaling pathway, such as *BMP2*, *BMP4*, *BMP7*, and *Samd2*, and expression levels of other related factors, such as *FGF7*, *CYP17*, *CXCL13*, *EDAR*, *TGFβ2*, *PI3KB*, and *Akt*, were also comparable.

### Shh signaling participates in the process of wool and active hair-follicle density increase through GLI2

*Sonic hedgehog* (*Shh*) signaling plays a critical role in hair-follicle development and skin cancer. Since we have previously confirmed the involvement of *Wnt/β-catenin* signaling, as a factor downstream of the *Wnt/β-catenin* signaling pathway, we are also curious about the role of the *Shh* pathway in the increase of wool and active hair-follicle density. The same method as before, we examined the transcription of *Shh*, *Gli1*, *Gli2*, and *Gli3*. As a result, *Shh* mRNA level was significantly lower in the KO than in the control group (*P* < 0.05), *Gli2* mRNA level was significantly higher in the KO group, and the expression levels of *Gli1* and *Gli3* had no significant change (Fig. 3e). As Mill et al.^33^ demonstrated that *Gli2* is the key mediator of *Shh* responses in the skin, and *Gli2* controls epithelial cell proliferation in embryonic skin through transcriptional regulation of cyclin D1 and D2, we believed that *Shh* signaling participated in the process of active hair-follicle density increase through *Gli2*. c-MYC, whose expression level was significantly higher in the KO group, could also be activated by *Shh*.

### Addition of DHT and finasteride to DPCs demonstrates the interaction between androgen and Wnt/β-catenin

**Fig. 4:**
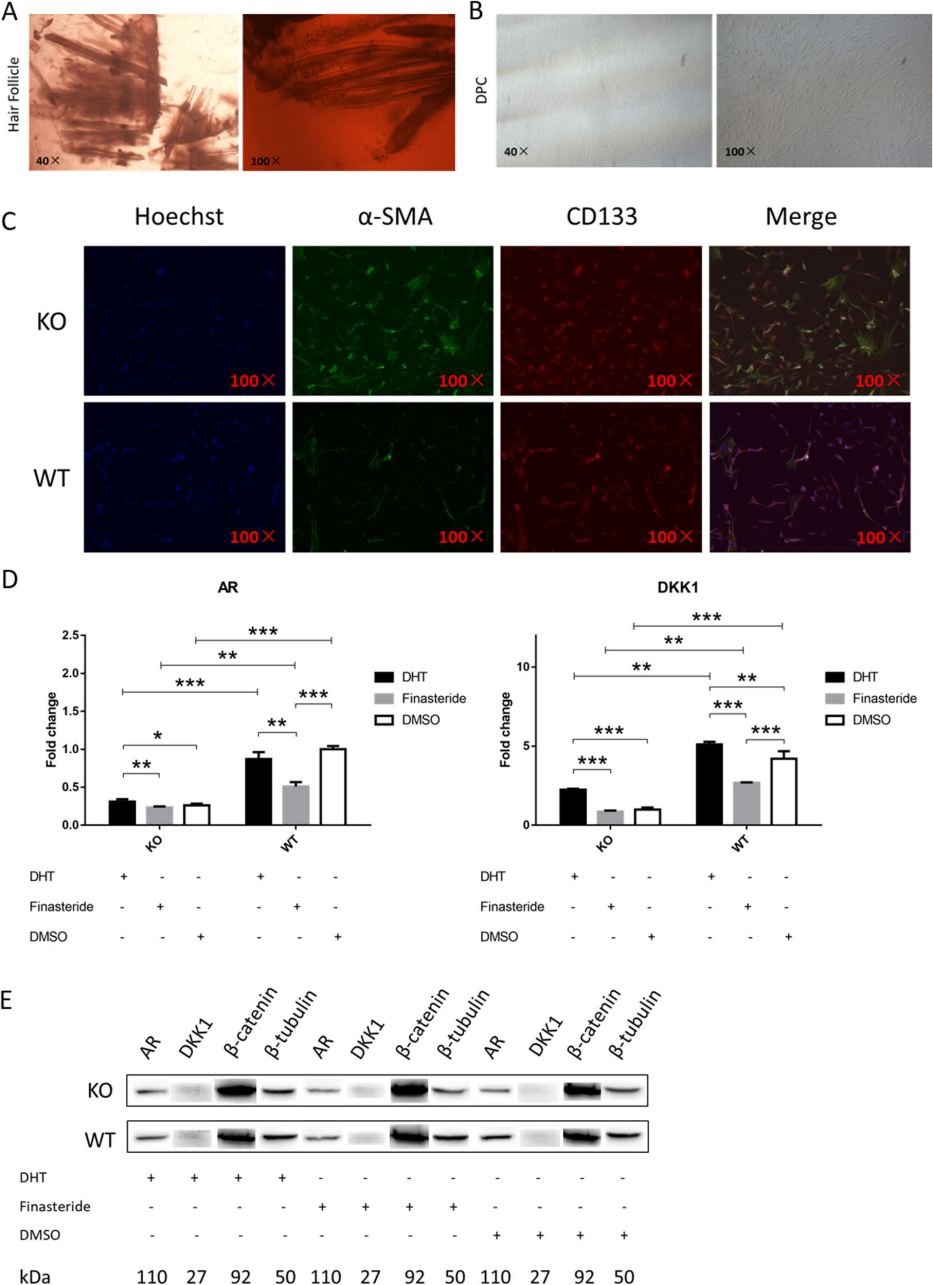
Addition of DHT and finasteride to DPCs demonstrates the interaction between androgen and Wnt/β-catenin. **a** Hair follicles of the KO and control group are obtained from skin tissue. **b** Dermal papilla cells are isolated and purified with a cloning loop. **c** The isolated dermal papilla cells were subjected to immunofluorescence staining. The positive α-SMA proved to be a dermal papilla cell, and CD133- positive proved that the cell was in the growth phase. **d** Changes in the relative expression levels of AR and DKK1 genes in DPCs of KO and the control group after the addition of DHT, finasteride, and DMSO, respectively. **e** Changes in the relative protein levels of AR and β-catenin in DPCs of KO and the control group after the addition of DHT, finasteride, and DMSO, respectively.

Since the crosstalk between androgen and *Wnt/β-catenin* pathway plays a pivotal role in the regulation of hair growth, the effects of DHT and finasteride on the levels of *AR* and *β-catenin* were examined in the DPCs. DPCs were obtained from sheep skin tissue by isolation and purification (Fig. 4a, b), and identified by immunofluorescence staining (Fig. 4c). The positive α-SMA proved to be DPCs, and CD133-positive proved that the DPCs were in the growth phase. When the DPCs were treated with 10^−7^ M DHT for 48 h, the level of *AR* in the DPCs of the KO group increased significantly, and the level of *DKK1* in the DPCs of the KO and control groups all increased significantly. When the DPCs were treated with 10^−5^ M finasteride for 48 h, the levels of *AR* and *DKK1* in the DPCs of the control group all decreased significantly (Fig. 4d). The concentrations of DHT and finasteride used were selected based on the results of previous experiments, as shown in Supplementary Fig. S5. Furthermore, regardless of the treatment, the expression levels of the *AR* and *DKK1* gene in the KO group were always significantly lower than those in the control group. Similar results were obtained by western blot analysis of AR, DKK1, and β-catenin (Fig. 4e).

## Discussion

CRISPR–Cas9 is an RNA-guided gene-editing tool that offers several advantageous characteristics in comparison with the conventional methods (e.g., zinc finger nucleases and transcription activator-like effector nucleases). Despite some limitations, such as efficient delivery and safety, CRISPR–Cas9 is still the most convenient tool for gene-editing purposes^34,35^. There were reports on the lengthening of the wool after the knockdown of the *FGF5* gene in sheep and goats^22,36,37^. However, there are no reports on the effects of *FGF5* gene on wool and hair-follicle density, except Wang et al.^37^. Here, we confirmed in Dorper sheep that the *FGF5* gene affects wool and active hair-follicle density, and that *FGF5* KO sheep have more fine wool and active secondary hair follicles.

Activation of the *Wnt/β-catenin* signaling pathway is important for the initiation and maintenance of hair morphogenesis^29,38^, and is critical for the maintenance of DPC-inductive properties required for hair-follicle regeneration and growth of the hair shaft^39,40^. Previous research had shown that in DP cells from AGA patients, the *Wnt/β-catenin* signaling pathway is negatively influenced by ligand-activated AR, inhibiting HFSC differentiation^28^. DHT-inducible DKK1 produced by balding DP cells promotes apoptosis of neighboring follicular keratinocytes in vitro, together with the higher expression of *DKK1* in balding compared with the haired scalp, suggesting that *DKK1* may be one of the key factors involved in the pathogenesis of AGA^41^. In the present study, we associate the phenotype of increased wool density with the phenotype of AGA, and tests of testosterone and DHT speculated that androgen-induced signaling pathways were highly likely to be involved. Thus, we focused on whether the *Wnt/β-catenin* signaling pathway was involved. The expression of β-catenin protein in the skin tissues of the KO group was significantly higher than in the control group. *DKK1* were significantly lower in the KO group in both the mRNA and protein levels. For further verification of the crosstalk between androgen and *Wnt/β-catenin* pathway, we added DHT, finasteride, and DMSO in the culture medium of DPCs to detect the expression of related genes. We explored the optimal concentration of DHT and finasteride in DPCs based on relevant reports^42^ (Supplementary Fig. S5). The results showed that when the DPCs were treated with 10^−7^ M DHT for 48 h, the level of *AR* in the DPCs of the KO group increased significantly, while the level of *AR* in the DPCs of the control group decreased significantly. We still have not got a reasonable explanation for the decrease of *AR* in the DPCs of the control group; however, it seems to be related to the amount of DHT contained in the sample itself. Since the control group itself contains more DHT, it requires more exogenous DHT to function. When the DPCs were treated with 10^−5^ M finasteride for 48 h, the levels of *AR* and *DKK1* in the DPCs of the control group all decreased significantly, while the levels of *AR* and *DKK1* in the DPCs of the KO group decreased without significance. This may be due to the fact that the expression level of SRD5A1 in the KO group is already very low, and finasteride has a limited inhibitory effect compared with the control group.

Besides, the relationship between *FGFR1* and androgen has been reported in studies of the transition of hormone-naive to castrate-resistant prostate cancer^43^. However, the relationship between FGFR1 and androgen in hair follicles needs further exploration and improvement.

**Fig. 5:**
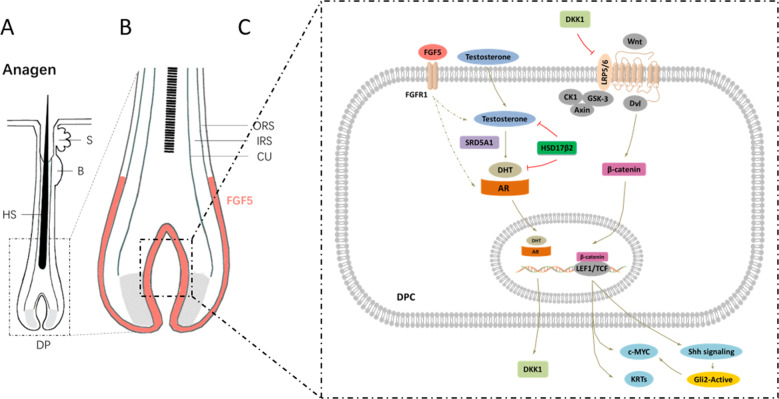
Diagram illustrating the signaling pathways involved in *FGF5* gene-editing sheep during anagen. **a** Schematic diagram of hair follicles during anagen. **b** An enlarged view of the DP area and the signaling pathways that occur in it. **c** The molecular mechanism of *FGF5* signaling cascade occurs in DPC, which changes the wool and the active hair-follicle density. The FGF5 protein secreted by the outer root sheath cells binds to its receptor FGFR1, causing changes in related signals in DPCs. Among them, the expression levels of SRD5A1 and HSD17β2 were changed, and the process of testosterone to DHT was also regulated. DHT can bind to AR and further into the nucleus, which positively regulates the expression of genes such as DKK1. As an inhibitor of the *Wnt* signaling pathway, DKK1 cascades the AR signaling pathway, and the *Wnt/β-catenin* signaling pathway to regulate the growth and development of hair follicles. In addition, the *Shh* signaling pathway and IRS-related *KRTs* and *C-MYC* also participate in the whole process as part of the downstream of the *Wnt/β-catenin* signaling pathway.

Finally, based on the increase in wool and active hair-follicle density in *FGF5* KO sheep, we first mapped out the main mechanism of *FGF5* function in anagen, including FGF5, FGFR1, androgen, AR, Wnt/β-catenin, c-MYC and KRTs, and Shh/GLI2 (Fig. 5), which helps to further understand the role of *FGF5* gene, and provide a treatment for androgen alopecia. Of course, we also tested the genes of other related signaling pathways (Supplementary Fig. S6). However, there are still more details that require further research and enrichment, such as the interaction of *Wnt-BMP*, *Noggin-Shh*, *Wnt-Notch*, and so on.

## Materials and methods

### Ethics statement

All animal experimental procedures in this study were approved and carried out in strict accordance with guidelines for the care and use of animals of the Animal Care and Use Committee at China Agricultural University (approval number CAU20140910-2). All surgical operations were performed under anesthesia.

The control group were siblings born at the same time as the experimental group. The investigator was blinded to the group allocation during the experiment.

### Production and screening of FGF5 gene-editing sheep

The process of production of *FGF5* gene-editing sheep is shown in Fig. 1a. Templates for Cas9 mRNA and ovis *FGF5* single-guide RNA transcription were constructed by our laboratory before. The target site in the ovis genome for *FGF5* sgRNA is shown in Fig. 1b.

The capacity of Cas9 mRNA and *FGF5* sgRNA for native gene disruption activity at its target locus was determined by DNA sequencing in microinjected individuals. The primers are as follows: forward, TGCAAGTTCAGGGAGCGATT and reverse, ATCCCTGTATGCACCAAGCA. Mutations were identified by alignment of sequenced alleles to the wild-type allele. DNAMAN software was used to perform multiple alignments.

### Off-target detection

To determine the site-specific cleavage of the CRISPR–Cas9 system in vivo, the potential off-target loci were searched using Cas-OFFinder (http://www.rgenome.net/cas-offinder). The selected potential off-target sites were initially PCR-amplified and then subjected to a DNA sequencing assay. The information of the off-target loci and primer pairs used is listed in Supplementary Table S2.

### Changes in protein sequences and structure prediction

The sequence of ovis FGF5 (NM_001246263.2) in NCBI (https://www.ncbi.nlm.nih.gov) was used. Changes in secondary structures were predicted by PSIPRED v3.3 (http://bioinf.cs.ucl.ac.uk/psipred). The Phyre2 server (http://www.sbg.bio.ic.ac.uk/phyre2/html/page.cgi?id = index) was used for homology modeling and fold recognition.

### Measurements of associated genes by real-time PCR

The total RNA was extracted from the skin of the KO and control groups using TRIzol reagent (Thermo Fisher Scientific, ShangHai). PrimeScrip RT reagent Kit with gDNA Eraser (Perfect Real Time) (Takara Biomedical Technology, Beijing) was used to obtain the cDNA. Real-time PCR was performed on ABI Stratagene Mx3000P instrument (Agilent Technologies, Santa Clara, CA, USA) using a TB Green Premix Ex Taq II (Takara Biomedical Technology, Beijing). The primer sequences used in this experiment are shown in Supplementary Table S3. The expression levels were analyzed using the 2^ΔΔCt^ method, and normalized against *GAPDH*. Each sample was run in triplicate.

### ELISA and western blotting analysis

For ELISA, skin tissue samples of the KO and control groups were obtained and homogenized by hand grinders, removing the supernatant after centrifugation. The ELISA procedure was performed as described in the product (TSZELISA, USA). The concentrations of androstenedione and testosterone in the skin tissue were measured using an androstenedione ELISA kit (Hermes Criterion Biotechnology, Vancouver, Canada) and a testosterone ELISA kit (Hermes Criterion Biotechnology, Vancouver, Canada) in accordance with the manufacturer’s protocol.

For western blotting, equal amounts of protein of sheep skin tissue or DPCs were resolved on 12% SDS-polyacrylamide gel (SDS-PAGE) and transferred to the polyvinylidene difluoride membrane (PVDF). After incubation with antibodies, protein bands were detected using ECL chemiluminescence (Thermo Fisher Scientific, USA). The primary antibodies were rabbit anti-FGF5 antibody (Thermo Fisher Scientific, USA), goat anti-SRD5A1 antibody (Abcam, UK), rabbit anti-androgen receptor antibody (Abcam, UK), goat anti-DKK1 antibody (Abcam, UK), beta-catenin polyclonal antibody (Proteintech, USA), c-MYC polyclonal antibody (Proteintech, USA), and beta tubulin monoclonal antibody (Proteintech, USA). The secondary antibodies were HRP-conjugated affinipure goat anti-rabbit IgG(H + L) (Proteintech, USA), HRP-conjugated affinipure rabbit anti-goat IgG(H + L) (Proteintech, USA), and HRP-conjugated affinipure goat anti-mouse IgG(H + L) (Proteintech, USA).

### Analytical procedures of wool phenotype of FGF5 KO sheep

The diameters of 200 randomly chosen fiber samples were measured using an Optic Fiber Diameter Analyzer (CU-6, Beijing United Vision Technical Company, Beijing, China). Photographs were documented for 48 h after shaving to provide a record of wool density. Images were captured with a handheld digital microscope—HotBeauty Skin tester Ht-B20S. Photographs were analyzed by using the ruler to correct the actual field of view, and the densities of coarse and fine wool were calculated separately.

### Sacpic of hair follicles

Sacpic is a histological method for determining the proportion of growing hair follicles in skin samples. Skin preparations of the KO and control groups were used to illustrate structural characteristics of follicles viewed in cross section at various stages of the hair cycle. Data from skin biopsies were also used to demonstrate quantitative estimation of follicle activity.

### DP cells’ acquisition and culture conditions

The skin tissues of the KO and control groups were digested with neutral protease (Gibco, Grand Island, NY, USA) at 37 °C for 2 h. The epidermis was carefully peeled off; the hair follicles were separately clustered from other tissues with a microscopic flaw. Excess tissues between hair follicles were removed by digestion with collagenase II (Gibco, Grand Island, NY, USA) at room temperature for about 30 min. Finally, the hair follicles were transferred to the culture dish; DP cells will slowly overflow in DMEM/F-12 (Dulbecco’s Modified Eagle Medium/Nutrient Mixture F-12), 20% (v/v) fetal bovine serum (FBS) supplemented with 100 units/ml penicillin and 0.1 mg/ml streptomycin (Invitrogen, Carlsbad, CA, and Sigma, St. Louis, MO, USA). DPCs were further purified and extracted using a cloning loop. For all experiments, cells were used between passage three and six.

The effects of DHT and finasteride on DPCs in the KO and control groups were also determined. DPCs (1.0 × 10^5^ cells/ml in 100-mm dishes) were seeded into 100-mm culture dishes. After a night of adherent growth, DPCs were treated with 10^−7^ M DHT and 10^−5^ M finasteride (Solarbio, Beijing, China) for the next 48 h, respectively. The media were changed every 24 h. After 2 days of conditioned culture, DPCs were collected for the extraction of total RNA and protein.

### Immunofluorescence analysis

DPCs were seeded in 96-well plates (Corning, USA) in each well. Cells were fixed with 4% paraformaldehyde for 20 min at room temperature, and then permeabilized with 0.3% Triton X-100 for 10 min at room temperature. They were washed three times with PBS for 5 min. After a 20-min incubation with immunol staining blocking buffer (Beyotime, Shanghai, China), cells were continuously incubated with mouse anti-SMA (1:100, Abcam) and rabbit anti-CD133 (1:100, Abnova)^44^ at 4 °C overnight. Then cells were washed three times with PBS followed by incubation with the secondary antibody (1:200, Proteintech) and Hoechst 33342 (1:1000) for 1 h. Ninety-six-well plates with the fixed cells were examined using ImageXpress (Molecular Devices).

### Statistical analysis

Statistical comparison of wool fineness and density was performed by the one-way ANOVA. Statistical analyses were performed using SAS release 9.2 (SAS Inst. Inc., Cary, NC).

## Supplementary information

Fig S1

Fig S2

Fig S3

Fig S4

Fig S5

Fig S6

Supplementary Figure legends

Supplementary Table S1

Supplementary Table S2

Supplementary Table S3
