# Supplementary figures and images for: Crosstalk between androgen and Wnt/β-catenin leads to changes of wool density in FGF5-knockout sheep
Source: Cell Death Dis. 2020 May 29;11(5):407. doi: 10.1038/s41419-020-2622-x (PMC7260202; doi:10.1038/s41419-020-2622-x)

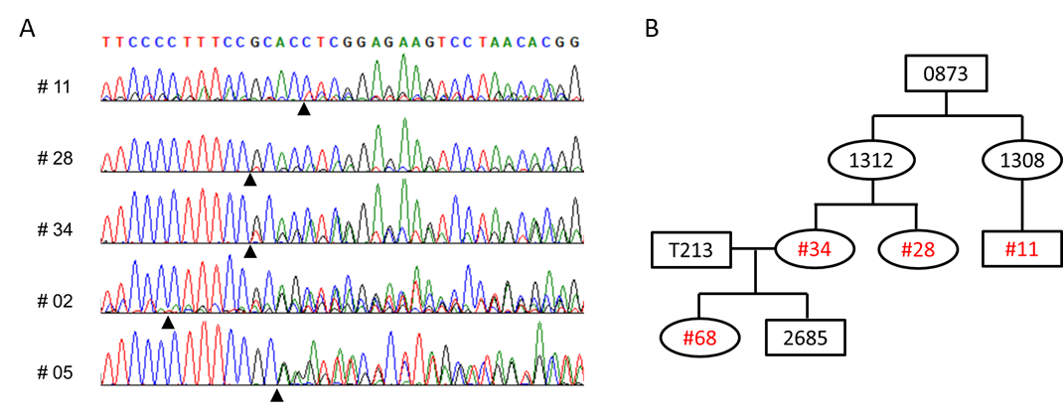

Supplement: Supplementary file 1 — Fig S1 [file 41419_2020_2622_MOESM1_ESM.tif]

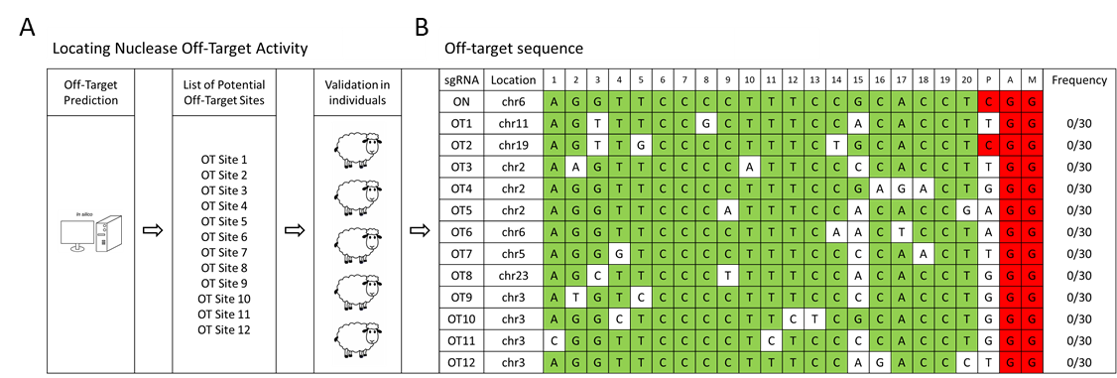

Supplement: Supplementary file 2 — Fig S2 [file 41419_2020_2622_MOESM2_ESM.tif]

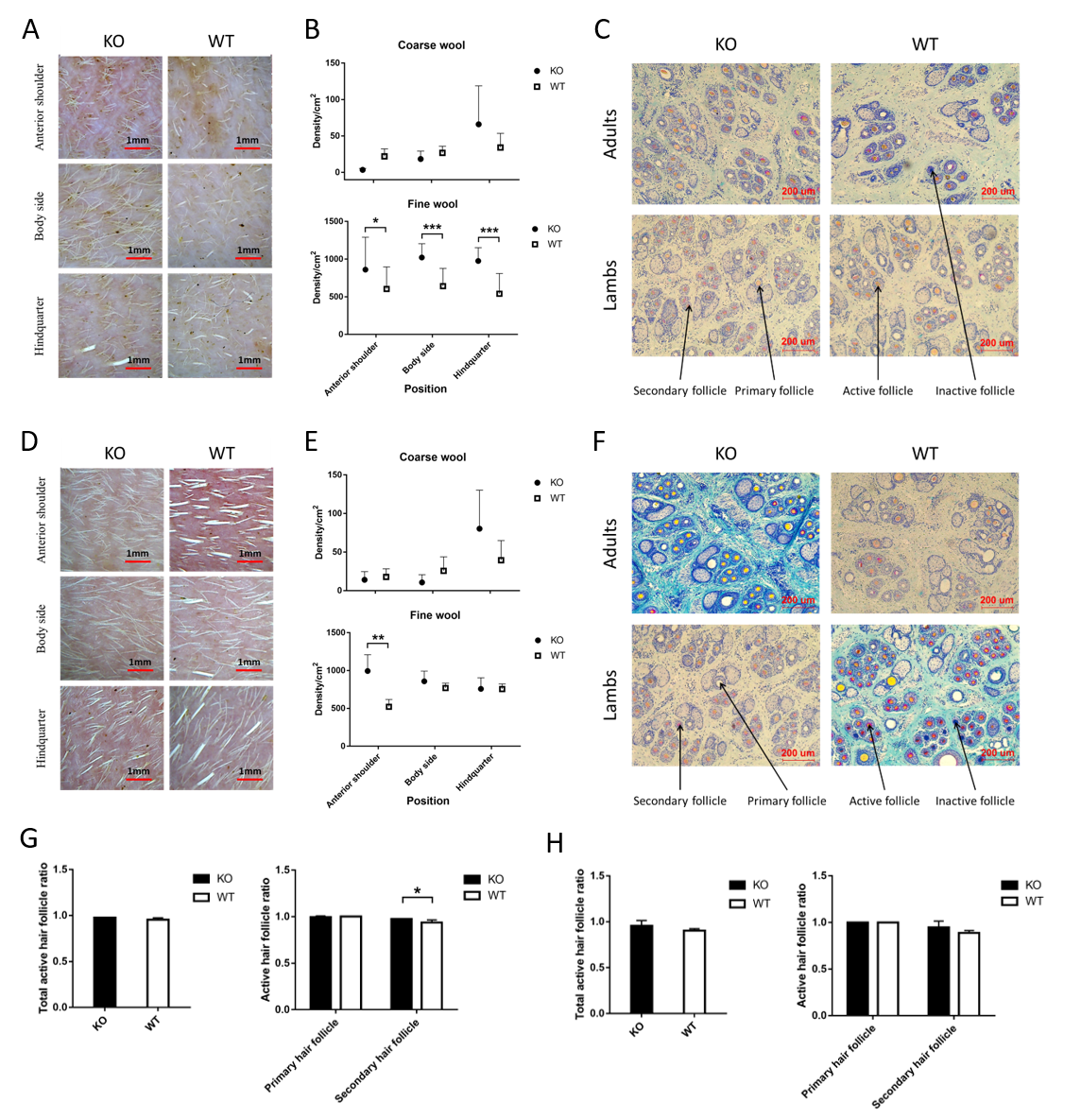

Supplement: Supplementary file 3 — Fig S3 [file 41419_2020_2622_MOESM3_ESM.tif]

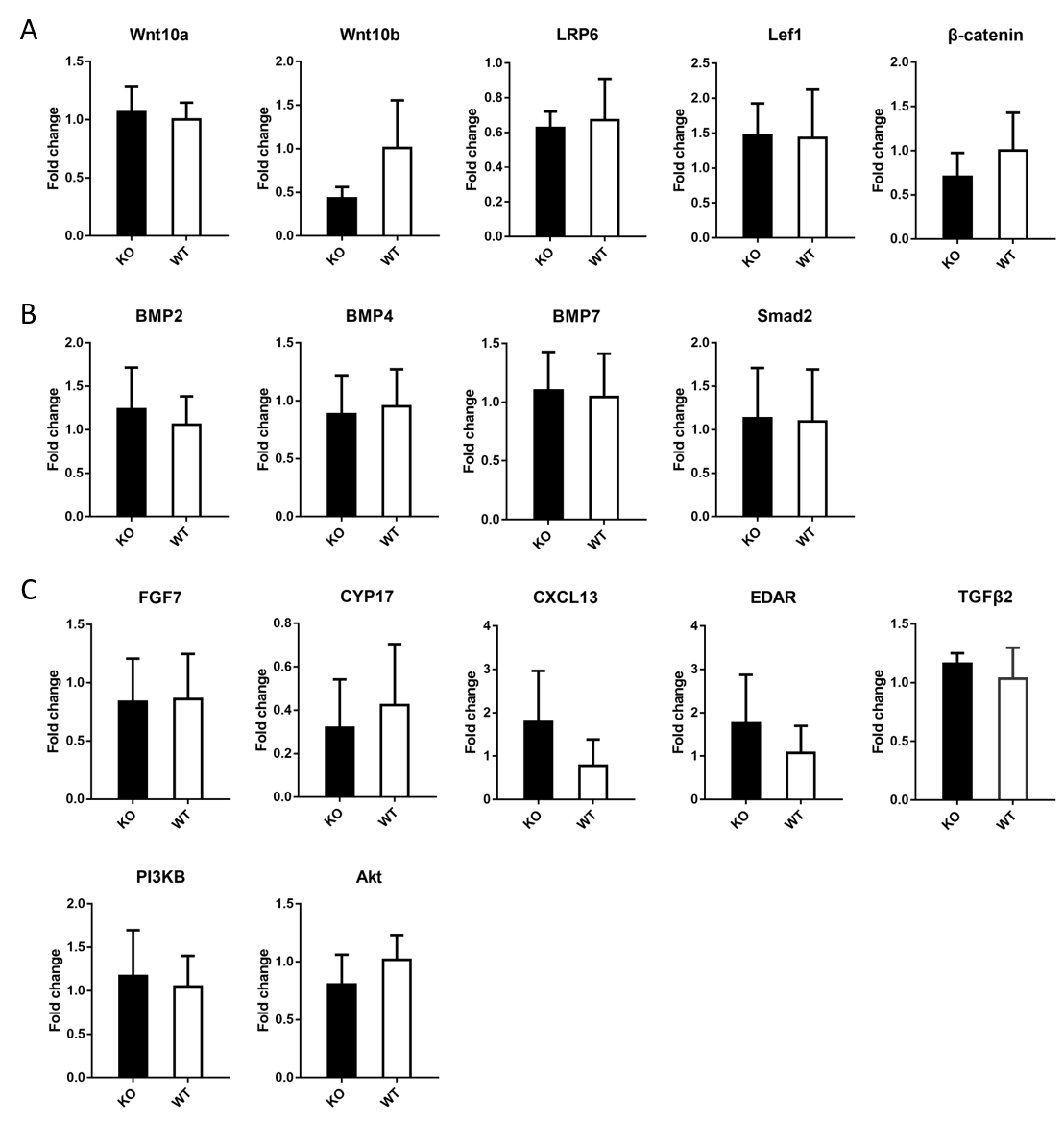

Supplement: Supplementary file 4 — Fig S4 [file 41419_2020_2622_MOESM4_ESM.tif]

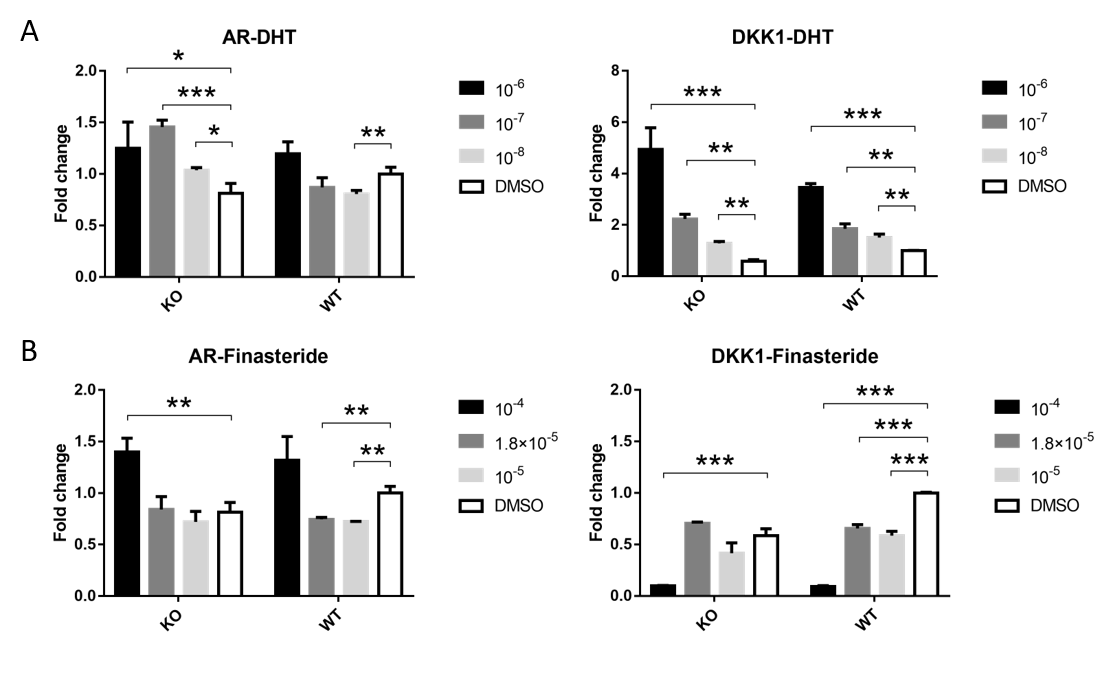

Supplement: Supplementary file 5 — Fig S5 [file 41419_2020_2622_MOESM5_ESM.tif]

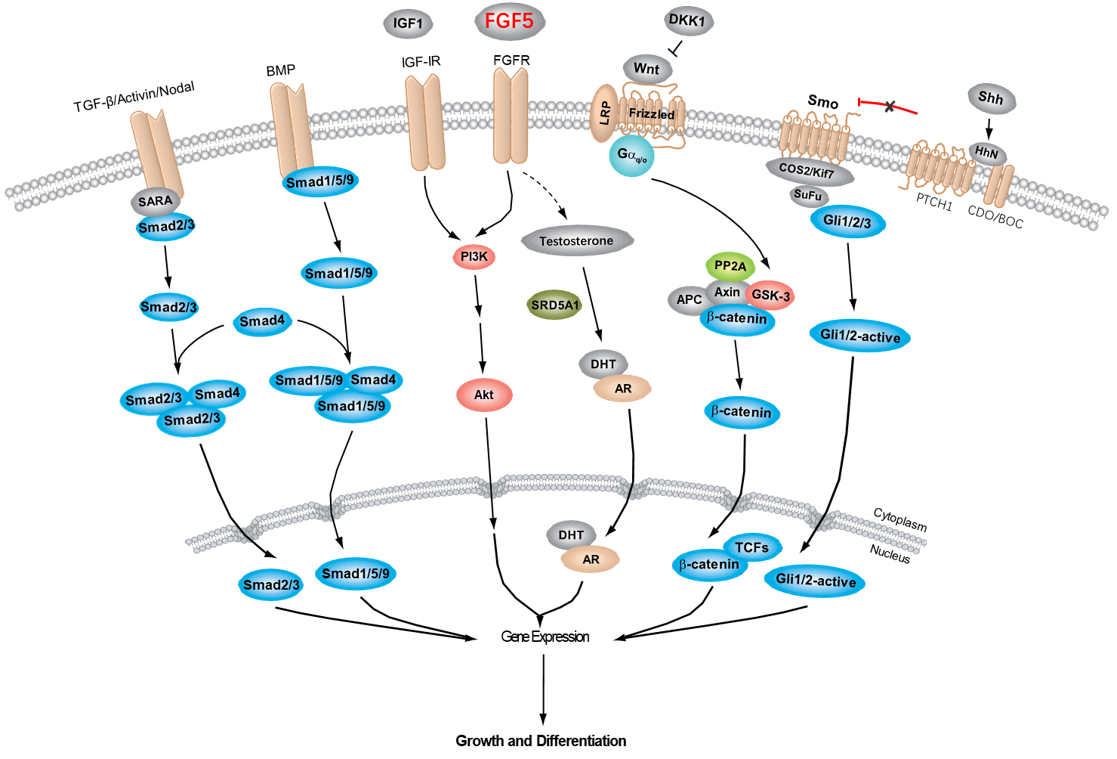

Supplement: Supplementary file 6 — Fig S6 [file 41419_2020_2622_MOESM6_ESM.tif]
