# Supplementary material for: Crosstalk between androgen and Wnt/β-catenin leads to changes of wool density in FGF5-knockout sheep
Source: Cell Death Dis. 2020 May 29;11(5):407. doi: 10.1038/s41419-020-2622-x (PMC7260202; doi:10.1038/s41419-020-2622-x)
Supplement: Supplementary file 7 — Supplementary Figure legends [file 41419_2020_2622_MOESM7_ESM.docx]

Fig S1. DNA sequencing peak maps of Founders and the phylogenetic map.

(A) DNA sequencing peak maps of Founders. The black triangle indicates where the mutation occurred. (B) The phylogenetic map of founders. Positive individuals are marked in red.

Fig S2. Locating nuclease off-target activity in the *FGF5* KO sheep.

(A) Off-target sites are predicted through silico techniques, which generate a list of potential off-target sites. (B) List of all mutations observed at an off-target site with three mismatches. Genomic DNA is harvested from *FGF5* KO sheep. The sites are amplified, tested. And no subset validated as bona fide off-target sites. On-target sites are in green, mismatches are backgroundless, PAM is in red. Note: Each off-target site was identified by sequencing of 30 TA clones, and the off-target efficiencies were calculated from the mutated ratio of TA clones at each off-target site.

Fig S3. Changes in sheep wool density and active hair follicle density after *FGF5* gene editing in catagen and telogen.

(A) and (D) are photographs of skin follicles at 48 hours after shaving in catagen and telogen, respectively. The actual area shown in the picture is 16 mm². (B) and (E) are the changes in coarse wool and fine wool density in the three parts of the KO group and control group in catagen and telogen, respectively. The density of fine wool in the KO group was significantly higher than that in the control group at all 3 sites in catagen, and at anterior shoulder in telogen, respectively. (C) and (F) are the sacpic staining of adult sheep and lamb skin sections in the KO group and control group. The ruler is marked in red in the lower right corner of the picture. Primary hair follicles, secondary hair follicles, active hair follicles, and inactive hair follicles are also labeled with arrows, respectively. (G) and (H) are changes of total active hair follicle ratio and active hair follicle ratio of coarse wool and fine wool between the KO group and control group. The statistical results in Figure B and E are from Figure A and D respectively, and the statistical results in Figure G and H are from Figure C and F respectively.

Fig S4. Changes in the expression levels of other related genes detected by q-PCR.

(A) Expression changes of other related genes in *Wnt* signaling in the skin tissue of KO group and control group. (B) Expression changes of other related genes in *BMP* signaling in the skin tissue of KO group and control group. (C) Expression changes of other related genes in the skin tissue of KO group and control group.

Fig S5. Changes in *AR* and *DKK1* expression after addition of different concentrations of DHT and finasteride in DPCs.

(A) Changes in *AR* and *DKK1* expression after addition of different concentrations of DHT in DPCs. (B) Changes in *AR* and *DKK1* expression after addition of different concentrations of finasteride in DPCs.

Fig S6. A summary of the signaling pathways involved in this study, including *Wnt/β-catenin* signaling pathway, *Shh* signaling pathway, *TGFβ* signaling pathway, *BMP* signaling pathway, and *IGF1/PI3K* signaling pathway.
