# Supplementary material for: Crosstalk between androgen and Wnt/β-catenin leads to changes of wool density in FGF5-knockout sheep
Source: Cell Death Dis. 2020 May 29;11(5):407. doi: 10.1038/s41419-020-2622-x (PMC7260202; doi:10.1038/s41419-020-2622-x)
Supplement: Supplementary file 8 — Supplementary Table S1 [file 41419_2020_2622_MOESM8_ESM.docx]

Table S1 Research on the accuracy of DNA sequencing

| Treatment | #68 | #68+2control | #68+3control | #68+4control | #68+5control |
| --- | --- | --- | --- | --- | --- |
| Editing efficiency | 50% | 16.7% | 12.5% | 10% | 8.3% |
| Sequencing peak | 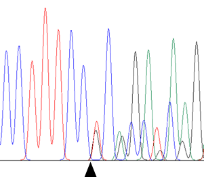 | 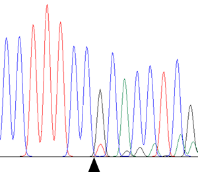 | 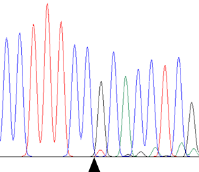 | 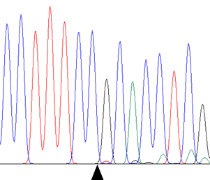 | 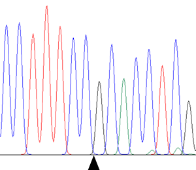 |

Note: #68 is a progeny produced by crossing #34 and a negative individual, and the heterozygosity rate of *FGF5* gene in which was 50%. The black triangle indicates where the mutation occurred.
