# Supplementary material for: Crosstalk between androgen and Wnt/β-catenin leads to changes of wool density in FGF5-knockout sheep
Source: Cell Death Dis. 2020 May 29;11(5):407. doi: 10.1038/s41419-020-2622-x (PMC7260202; doi:10.1038/s41419-020-2622-x)
Supplement: Supplementary file 9 — Supplementary Table S2 [file 41419_2020_2622_MOESM9_ESM.docx]

Table S2 Primers for amplification of each off-target region

| Off-target sites | Location | Primer sequence (5’–3’) | Product size (bp) | T_m_ (°C) |
| --- | --- | --- | --- | --- |
| OT1 | Chr11 | Forward: GCACTGCGTTGCTGATGTTA  Reverse: GTCACCACGGGATACTCAAG | 515 | 58 |
| OT2 | Chr19 | Forward: AGGGGTTCACAGTCAGCTTG  Reverse: TTTCTGGCACGGTTGTGAGG | 444 | 58 |
| OT3 | Chr2 | Forward: AAGCCAATAGTTGATAAACCAGCA  Reverse: ATCTCATGACCTCTGGGGCAG | 582 | 54 |
| OT4 | Chr2 | Forward: CTGAGTAGGGAGTCGGCTTT  Reverse: GGCCAGGTTCTTCATGAGGTT | 157 | 58 |
| OT5 | Chr2 | Forward: CAGTGTGGTGGAGCCTTCTT  Reverse: CTCCACATGATTCTGATGTCT | 350 | 58 |
| OT6 | Chr6 | Forward: CCCCCACGGGGTTATCTCTA  Reverse: AGGGGGCTAGAAGCACTCTT | 875 | 58 |
| OT7 | Chr5 | Forward: CAAGGCTGAGGATTTCTGAT  Reverse: AGTGTGGCATCCATTGGTGG | 359 | 58 |
| OT8 | Chr23 | Forward: GCTTTGGGACATTCATCGGC  Reverse: CATGGGACGCTCTGTACCAA | 602 | 58 |
| OT9 | Chr3 | Forward: GATGGAGGAGAATCCGCTCTCA  Reverse: GGAGAGTCTGGGTCACTTGCT | 503 | 58 |
| OT10 | Chr3 | Forward: TGGATGATCCCAAACGGCAA  Reverse: CTGAAGGTTGTGAACCCCAG | 256 | 58 |
| OT11 | Chr3 | Forward: GGTACCTGAGTCATACAGCTAG  Reverse: TATACACTCCTTAGAACTCATC | 586 | 54 |
| OT12 | Chr3 | Forward: AGTCCTGCTCTCGTGCACAGA  Reverse: TCTGACCTCAGACAGACACTC | 477 | 58 |
