# Supplementary material for: Crosstalk between androgen and Wnt/β-catenin leads to changes of wool density in FGF5-knockout sheep
Source: Cell Death Dis. 2020 May 29;11(5):407. doi: 10.1038/s41419-020-2622-x (PMC7260202; doi:10.1038/s41419-020-2622-x)
Supplement: Supplementary file 10 — Supplementary Table S3 [file 41419_2020_2622_MOESM10_ESM.docx]

Table S3 Primers for qPCR

| Genes | Accession | Primer sequence (5’–3’) | Product size (bp) | T_m_ (°C) |
| --- | --- | --- | --- | --- |
| *FGF5* | NM_001246263.2 | Forward: CACGTCTCTACCCACTTTCTG  Reverse: TTAGGACTTCTCCGAGGTGC | 143 | 60 |
| *FGFR1* | XM_015104616.1 | Forward: CAAACCAAACCGTATGCCCG  Reverse: ACCTTGTAGCCTCCGATCCT | 192 | 60 |
| *SRD5A1* | XM_015101349.1 | Forward: AACCAGGGGAGACTGGCTAT  Reverse: CAAAAGCCCAGCCCTGAATG | 135 | 60 |
| *AR* | NM_001308584.1 | Forward: GGTGAGCAGAGTGCCCTATC  Reverse: AGTCGATGGGCAGAACATGG | 131 | 60 |
| *DKK1* | XM_012102454.2 | Forward: CTATGTGCTGCCCTGGGAAT  Reverse: GATGAGCGAAGACAGACGGT | 208 | 60 |
| *Shh* | XM_012138642.2 | Forward: TGAACGCCTTAGCCATCTCC  Reverse: GGTCCGAGGTGGTGATGTC | 141 | 60 |
| *HSD17β2* | NM_001267882.1 | Forward: CCAACTCGCAGGAGATACAAG  Reverse: GCTGAAATGCCTTCGTGACTT | 195 | 60 |
| *Gli1* | XM_015094784.1 | Forward: GCCACATATGGACCTGGCTT  Reverse: TTTGTTGGCCCCTACTGCAA | 102 | 60 |
| *Gli2* | XM_012140342.2 | Forward: AACACATCCCGCCATCTCTG  Reverse: AGAAACGCGACACATCCACT | 236 | 60 |
| *Gli3* | XM_012113061.2 | Forward: ATGTCACCAAGAAGCAGCGA  Reverse: ACTTGGAGGCATTCTTCCCG | 166 | 60 |
| *β-catenin* | NM_001308590.1 | Forward: GGGAGTCCGCATGGAAGAAA  Reverse: GCAGCTGCACAAACAATGGA | 122 | 60 |
| *KRT25* | NM_001009739.1 | Forward: GAGAGCGATGTCAATGGGCT  Reverse: GTTCAGCAGAACCGTGAGGT | 213 | 60 |
| *KRT27* | NM_001114763.2 | Forward: AATTTGGGCCTGGTTCCTGC  Reverse: GCTGTGAGCCTTGCATTGTC | 145 | 60 |
| *KRT28* | XM_004012873.3 | Forward: CTGGATCTTGCCGTGGACTT  Reverse: ACTCGCCGTAAGCCATTGAT | 217 | 60 |
| *KRT71* | NM_001280716.1 | Forward: GGATAACAACCGCGACCTGA  Reverse: GGCAGTCTCCAGGTTGGAAG | 259 | 60 |
| *GAPDH* | NM_001190390.1 | Forward: CTGACCTGCCGCCTGGAGAAA  Reverse: GTAGAAGAGTGAGTGTCGCTGTT | 149 | 60 |
| *FGF5s* | NM_001291082.1 | Forward: AGGAAGCGGCTTGGAGCAGA  Reverse: TGTAAATTTGGCTTAACATAT | 158 | 60 |
| *Wnt10a* | XM_012113696.2 | Forward: AACATCCTTCGGCAGACACG  Reverse: TCACTTGCAGACACTGACCC | 111 | 60 |
| *Wnt10b* | XM_004023070.4 | Forward: CTCAAGCGCGGTTTCCGAG  Reverse: GTTTCGCCCTCAGTCGATCC | 154 | 60 |
| *LRP6* | XM_004006855.3 | Forward: AACAGACGGGACTTGCGATT  Reverse: AATCACATGCCAGCCCATCA | 232 | 60 |
| *Lef1* | XM_004009634.3 | Forward: CCCCACCTCTTGGCTGGTTTTCTCA  Reverse: TTGGCTCCTGCTCCTTTCTCTGTTC | 177 | 60 |
| *BMP2* | XM_004014353.4 | Forward: GGAGAAGGAAGAGGCGAAGG  Reverse: GGTCGACCTTTAGGAGACCG | 139 | 60 |
| *BMP4* | XM_015096688.1 | Forward: CACCACGAAGAACATCTGGAGAACA  Reverse: CGGCAGACGAGATCACCTCATTC | 109 | 60 |
| *BMP7* | NM_001308564.1 | Forward: GCTATACGTCAGCTTCCGGG  Reverse: TGATGAAGTGTACCAGCGTCT | 158 | 60 |
| *Smad2* | XM_027960885.1 | Forward: GCCGCCCGGAGGTTAGAT  Reverse: TCCAGATCCACCAGCTGACT | 142 | 60 |
| *FGF7* | NM_001009235.2 | Forward: ATGCAAAGAAAGAATGTAACGAAGA  Reverse: CCCTCTTACTGGAACCCCCT | 146 | 60 |
| *CYP17* | XM_012102863.2 | Forward: GATCGTGGCCTACCTGCTAC  Reverse: TCGCCAATGCTGGAGTCAAT | 210 | 60 |
| *CXCL13* | XM_012122842.1 | Forward: ACGGTGTTCTGGAGACCAAT  Reverse: TCCTTTTGGCTTGAGGGTTCA | 189 | 60 |
| *EDAR* | XM_015094371.1 | Forward: TGCTATCGCTGGTCCACCT  Reverse: ACCGCCTTCTCCGAGTTGTA | 205 | 60 |
| *TGFβ2* | XM_004013602.3 | Forward: ACATGCCGTCCTTCTTACCC  Reverse: GGATGGCATCAAGGTACCCA | 120 | 60 |
| *PI3KB* | XM_004003302.4 | Forward: GTATCTTAAGGACTCTCTTGCCT  Reverse: AACACATTAGGAGCAAAGGCTG | 174 | 60 |
| *Akt* | NM_001161857.1 | Forward: GAGACGATGGACTTCCGGTC  Reverse: CTCATTCATGGTCACGCGGT | 102 | 60 |

Note: All primers were synthesized by SanGon (Shan Hai, China)
